# Genetic loci, rs17817449 and rs6567160, known for obesity and the risk of stroke events among middle-aged and older Chinese people

**DOI:** 10.3389/fneur.2022.1036750

**Published:** 2022-12-02

**Authors:** Qiong-Qiong Zhong, Feng Zhu

**Affiliations:** ^1^Department of Science and Education, Guangzhou Twelfth People's Hospital, Guangzhou, China; ^2^Department of Public Health and Preventive Medicine, School of Medicine, Jinan University, Guangzhou, China

**Keywords:** genetic loci, single-nucleotide polymorphism, obesity, stroke, risk, middle-aged to older

## Abstract

**Background:**

Fat Mass and Obesity-Associated (FTO) and the Melanocortin-4 Receptor (MC4R) genes are strongly associated with obesity, an established risk factor for stroke. We aimed to assess the associations between rs17817449 at the *FTO* and rs6567160 at the *MC4R* and the risk of stroke events in middle-aged and older Chinese people.

**Materials and methods:**

Study data were obtained from the Guangzhou Biobank Cohort Study; a total of 148 participants with a self-reported history of stroke and an equal volume of age- and sex-matched participants were selected as the cases and the controls in a case-control study; a total of 13,967 participants at the first follow-up and all participants with fatal stroke (up to April 2021) were included in a retrospective cohort study. Conditional logistic regression and the Cox proportional hazards regression analyses were used to assess the associations of the two genetic loci with the risk of stroke events.

**Results:**

After adjusting for age, sex, education, job, smoking, alcohol consumption, body mass index, physical activity, hypertension, diabetes, and dyslipidemia, rs17817449 and rs6567160 shared minor alleles G and C, respectively, in the case-control analyses. The genotypes GG+GT of rs17817449 at the *FTO* were significantly associated with a decreased risk of fatal stroke occurrence, with fatal all strokes having an adjusted hazard ratio (aHR) of 0.71 (95% confidence intervals (CI) 0.52-0.97, *P* = 0.04) and fatal ischemic stroke having an aHR of 0.64 (95% CI 0.41–1.00, *P* = 0.05), when the genotype TT was taken as a reference and a series of multiplicities were adjusted; the risk of fatal all strokes was lowered by dyslipidemia (aHR = 0.63, 95% CI 0.39–1.00, *P* = 0.05) and non–diabetes (aHR = 0.68, 95% CI 0.46–0.99, *P* = 0.049) in the retrospective cohort analyses. Significances were observed neither in the associations between rs6567160 and the risk of stroke events nor in an interaction between rs17817449 and rs6567160 in the two-stage analyses.

**Conclusion:**

The G allele of rs17817449 at the *FTO*, not rs6567160 at the *MC4R*, was associated with a decreased risk of fatal stroke occurrence; its functional role in stroke should be explored in relatively healthy middle-aged to older Chinese people.

## Introduction

Stroke is the second leading cause of death and the third leading cause of disability, with 70% of stroke cases and 87% of both stroke-related deaths and disability-adjusted life years occurring in low- and middle-income countries worldwide (1). Considering that China accounts for a fifth of the global population and that over 2 million new people in the country are diagnosed with stroke every year, the whole society faces a disproportionate share of this burden (2).

Genetic variation of obesity on stroke events has been widely explored in the last decade. Fat Mass and Obesity-Associated (*FTO*) is the first susceptibility gene for obesity identified by the Gene-Wide Association Studies (GWAS). It was related to a series of diseases, including diabetes, heart failure, cancer, and coronary heart disease (CHD) (3). Several genetic variants of the *FTO*, such as rs17817449, have also been shown to confer a very significant risk for obesity (4). Its risk alleles within the 47 kb linkage disequilibrium (LD) block on the *FTO* sections of intron one and exon two are associated with obesity (5), wherein energy homeostasis and eating behavior are controlled (6). The Melanocortin-4 Receptor (*MC4R*) has been associated with a balance between feeding and energy (7). As a G-protein-coupled melanocortin receptor in the mammalian genome in the Central Nervous System (8), the *MC4R* is activated principally by alpha-melanocyte-stimulating hormone. It then leads to neuronal depolarization and action potential firing. Central administration of the *MC4R* agonists promotes satiety, energy expenditure, and weight loss, whereas the antagonists increase food intake, energy conservation, and weight gain (9). Mutations in the *MC4R* could induce obesity syndromes (10), wherein rs6567160 may exert pleiotropic effects on both fat and lean body mass (11). The polymorphisms in the *FTO* and the *MC4R* were linked to overweight or obesity in children and adolescents (12). The effects, combined with the *MC4R* variants, hypertension, and smoking, were significantly associated with the risk of large-arterial atherosclerotic (LAA) stroke in China (13). However, the relationships between the *MC4R* and the *FTO* and the risk of a fatal stroke remain largely unknown. Our previous research screened a series of loci related to obesity through literature searches and analyses. We carried out a series of tests for single-nucleotide polymorphism (SNP) in a large-scale population from the Guangzhou Biobank Cohort Study (GBCS) (14, 15). Considering the most well-known associations between obesity and strokes, we aimed to assess the potential genetic roles of the *MC4R* and the *FTO* in populations susceptible to stroke. We also tested the sole and combined genetic effects on stroke events in relatively healthy middle-aged to older Chinese people.

## Materials and methods

### Study design, data source, and participants

A case-control and retrospective cohort study was conducted in the GBCS from 30 September 2003 to 13 April 2021. All participants in this study were recruited during the first follow-up period the details of which were reported in an earlier study (16). Briefly, the GBCS, initiated by the Guangzhou Twelfth People's Hospital and the Universities of Hong Kong and Birmingham, is an ongoing prospective cohort to examine genetic, lifestyle, occupational, and environmental factors. A total of 30,430 permanent residents aged 50 years or older in Guangzhou were recruited from September 2003 to February 2008, and a total of 18,158 participants completed the first follow-up from March 2008 to December 2012, in which 16,465 participants underwent genetic testing.

**Figure 1:**
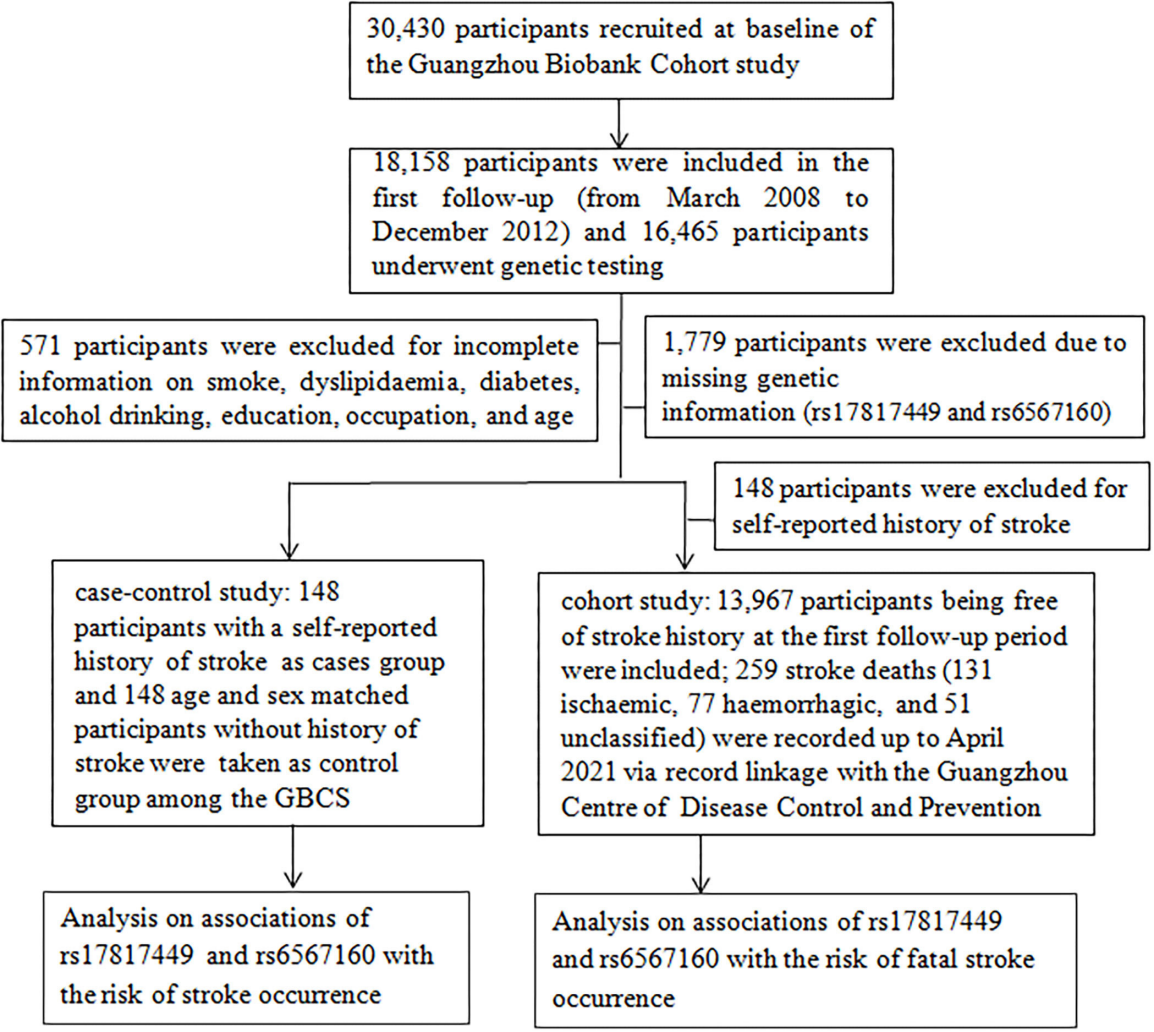
A flow diagram of the selected participants and study design for the analyses of this study.

We conducted a two-stage analysis with 16,465 participants. In a case-control study, 571 participants were excluded due to incomplete information on smoking, dyslipidemia, diabetes, alcohol consumption, education, occupation, and age; 1,779 participants were excluded due to missing genetic information; eventually, 148 participants with a self-reported stroke history (up to December 31, 2012) and an equal volume of participants without a history of stroke were recruited, respectively, as the cases and the controls (paired up with gender consistency and age within 1 year). In a retrospective cohort study, 148 participants with a self-reported stroke history were excluded; a total of 13,967 participants without stroke at the first follow-up were included; 259 stroke deaths (131 ischemic, 77 hemorrhagic, and 51 unclassified) were regarded as censored at the date of death (17) and eventually recorded after a mean of 10.7 years (SD = 2.03) of follow-up (up to 19 April 2021; Figure 1).

### Measurements

A computer-based, standardized structured questionnaire, including socioeconomic status, family and personal disease histories, and lifestyle, was administered by full-time trained nurses and face-to-face interviews in appropriate regional dialects. Smoking status was classified as “currently smoking” (people who smoked one cigarette daily for more than half a year at the time of the survey), “ever smoking” (people who smoked daily for at least 6 months during their lifetime but did not smoke around the time of the survey), and “never smoker” (people who never smoked at the time of the survey or in the past) (16; S. S. 13). Alcohol consumption was categorized into three categories: never drinking (did not drink any alcoholic beverage throughout their life), ever drinking (had abstained from alcohol for at least 1 year), and current drinking (drank one time a week or only on special occasions) (18, 19). Hypertension was defined as systolic and diastolic blood pressure (SAP and DB)≥140/90 mmHg or medication treatment for hypertension (20). After an initial 5-min rest, seated blood pressure was measured three times at 1–5 min intervals; the SAP and the DB were calculated as the average of the last two measurements (21). Diabetes was defined as fasting glucose≥7.0 mmol/L and/or an anti-diabetic medication or a previous diagnosis of diabetes (22, 23). The body mass index (BMI) was calculated as weight (kg)/standing height (m^2^) and regarded as obesity (BMI≥28 kg/m^2^), overweight (27.9 kg/m^2^> BMI≥24 kg/m^2^), normal weight (23.9 kg/m^2^≥BMI≥18.5 kg/m^2^), and underweight (BMI < 18.5 kg/m^2^) according to the criteria in the Asian populations (24). The specific physical activity classification was active, moderate, and inactive, according to the Chinese version of the International Physical Activity Questionnaire (IPAQ) (17, 25). Triglycerides (TG), total cholesterol (TC), low-density lipoprotein cholesterol (LDL-C), and high-density lipoprotein cholesterol (HDL-C) were determined at a laboratory in the Guangzhou Twelfth People's Hospital (15).

### Genotyping

Plasma aliquots were stored at −80°C. Deoxyribonucleic acid (DNA) was extracted from the whole peripheral blood sample using the proteinase K-phenol chloroform method. The SNPs were analyzed by a reputable commercial company (Beijing Capital Bio Corporation, Beijing, China) using a mass array system (Sequined, San Diego, CA, USA). After completing the polymerase chain reaction (PCR) amplification, the primer extension products were analyzed through the chip-based Matrix-Assisted Laser Desorption/Ionization-Time of Flight Mass Spectrometry (MALDl-TOF MS) and the SpectrolTYPER 4.0 software, which performs the genotype calling automatically using a set of digital filters optimized for the mass spectra of oligonucleotides (26–28).

### Study outcomes

Stroke occurrence was chosen as the primary outcome in this case-control study, and its underlying causes were obtained from participants with a self-reported history of stroke. This retrospective cohort study chose fatal stroke occurrence as the primary outcome. Its underlying causes were obtained using a record linkage with the Guangzhou Center for Disease Control and Prevention (GZCDC). Death causes were coded according to the 10th revision of the International Classification of Diseases (ICD) as follows: I60–I69 for stroke; I60.0–I62.9 and I69.0–69.2 for hemorrhagic stroke; I63–I63.9 and I69.3 for ischemic stroke; and the other codes for unclassified stroke. Additionally, five prominent clinicians from the Guangzhou Twelfth People's Hospital, the Universities of Hong Kong, China, and Birmingham, UK, cross-checked previous medical histories and performed a verbal autopsy.

### Statistical analysis

Continuous variables are presented as mean ± SD, and their normality was assessed using skewness, kurtosis, and the P-P plot. Categorical variables were described by frequency and percentage. The tests for differences in means and proportions were performed using the *t*-test and the Chi-square test. The Hardy–Weinberg Equilibrium (HWE) was used to assess genotype frequencies with the Chi-square test in groups (29). The odds ratio (OR) values and 95% confidence intervals (CIs) were calculated to evaluate the association between the two SNPs and stroke events, and a *P* < 0.025 (equivalent to a *P* < 0.05 after adjusting for two SNPs in independent tests by the Bonferroni correction) was defined as having statistical significance (30) when conditional logistic regression analyses were conducted in a case-control study. The hazard ratio (HR) values and 95% CIs were conducted in a retrospective cohort study; all *P*-values were two-sided, and a *P* < 0.05 was defined as statistical significance (17) when the Cox regression analyses were conducted as follows: Model A was a crude model; Model B was adjusted for age, sex, education, job, smoking, alcohol consumption, BMI, and physical activity; and Model C was adjusted for hypertension, diabetes, dyslipidemia, and the confounders in Model B. A stratified analysis was also run between the two SNPs and stroke in four different subgroups (hypertension, dyslipidemia, physical activity, and diabetes). Due to a small sample size for the genotype GG of rs17817449 and the genotype CC of rs6567160, the G or the C dominant model was used in stratified analyses, and the genotype TT was taken as a reference. The gene–gene interaction was performed using the Generalized Multifactorial Dimensionality Reduction (GMDR) method (19), wherein balanced accuracy testing scores between 0.50 (model prediction results are no better than chance) and 1.00 (perfect model prediction results) are indicative of the extent of the case-control status (30, 31). All analyses were performed using IBM SPSS Statistics for Windows (Version 22.0, Armonk, NY, USA).

## Results

### Case-control study

A total of 148 participants with a self-reported history of stroke and an equal volume of age- and sex-matched participants without a history of stroke were included as the cases and the controls. Age, the only continuous variable, was normally distributed (skewness value = −0.045 (standard error = 0.142), Z-score = −0.316; kurtosis value = −0.549 (SE = 0.282), Z-score = −1.946; the P–P diagram conforms to a normality distribution). Compared to the controls, the cases had more patients with hypertension (*P* < 0.001) and diabetes (*P* = 0.04) but fewer patients with manual occupation (*P* < 0.001) and who consume alcohol (*P* < 0.001) (Table 1).

**Table 1:** Baseline characteristics of the study participants (*n* = 296).

**Variable**	**Case group (*n* = 148)**	**Control group (*n* = 148)**	***P*** **value**
Age (years)	68.60 ± 6.91	67.63 ± 6.93	0.23
Sex, men, n (%)	61 (41.2)	61(41.2)	1.00
**Education, n (%)**			
Primary or below	58 (39.2)	70 (41.3)	0.36
Middle school	76(51.4)	65 (43.9)	
College or above	14 (9.5)	13 (8.8)	
**Occupation, n (%)**			
Manual	71(48.0)	95 (64.2)	<0.001^*^
Non-manual	54 (36.5)	49 (33.1)	
Other	23 (15.5)	4 (2.7)	
**Smoking, n (%)**			
Never	114 (77.0)	105 (70.9)	0.14
Ever	25 (16.9)	24 (16.2)	
Current	9(6.1)	19 (12.8)	
**Alcohol consumption, n (%)**			
Never	78 (52.7)	31 (20.9)	<0.001^*^
Ever	3 (2.0)	1 (0.7)	
Current	67 (45.3)	116 (78.4)	
**Physical activity**, ***IPAQ*****, n (%)**			
Inactive	3 (2.0)	4 (3.5)	0.66
Moderate active	51 (34.5)	44 (29.7)	
Active	94(63.5)	100 (67.6)	
**BMI (Body mass index, kg/m** ^**2**^ **), n (%)**			
<18.5	7(4.7)	9(6.1)	0.42
18.5–23.9	60 (40.5)	72 (48.6)	
24–27.9	66(44.6)	53 (35.8)	
≥28	15 (10.1)	14 (9.5)	
Hypertension, n (%)	124 (83.8)	83 (56.1)	<0.001^*^
Diabetes, n (%)	41 (27.7)	25 (17.6)	0.04^*^
Dyslipidemia, n (%)	94 (63.5)	85 (57.4)	0.28
**Rs17817449**			
GG	4 (2.7)	1 (0.7)	0.38
GT	30 (20.3)	33 (22.3)	
TT	114 (77.0)	114 (77.0)	
**Rs6567160**			
CC	3 (2.0)	3 (2.0)	0.87
CT	38 (25.7)	42 (28.4)	
TT	107 (72.3)	103 (69.6)	

* *P* < 0.05.

The distributions of rs17817449 and rs6567160 matched the HWE (*P*>0.05) and the minor allele frequencies (MAF) in the two SNPs by more than 0.01 in the cases and the controls (Table 2), wherein rs17817449 and rs6567160 shared minor alleles G and C, respectively. However, significance was observed neither in the association between rs17817449 and rs6567160 with the risk of stroke occurrence in three models (Table 3) nor in the interaction between rs17817449 and rs6567160 (Table 4).

**Table 2:** Allele distributions of rs17817449 and rs6567160 (*n* = 296).

**SNP ID**	**Position**	**Alleles (minor/major)**	**MAF (case, %)**	**MAF (control, %)**	***P*** **_HWE_**
Rs17817449	16:53779455	G/T	6.4	5.9	0.40
Rs6567160	18:60161902	C/T	7.4	8.1	0.59

**Table 3:** Gene-gene analyses by the GMDR method (*n* =296).

**Model combination**	**Training balance accuracy**	**Testing balance accuracy**	**Cross-validation consistency**	***P*** **value**
Rs17817449 rs6567160^A^	0.5270	0.4207	10/10	1.00
Rs17817449 rs6567160^B^	0.5452	0.4348	10/10	0.9453
Rs17817449 rs6567160^C^	0.5317	0.4130	10/10	0.9990

A : Model A: crude HR;

B : Model B: Adjusted for age, sex, education, job, smoking, alcohol consumption, BMI, physical activity;

C : Model C: Adjusted for age, sex, education, job, smoking, alcohol consumption, BMI, physical activity, hypertension, diabetes, and dyslipidemia.

**Table 4:** Genotype distributions and associations between rs17817449 and rs6567160 with the risk of all strokes (*n* =296).

**Genotype**		**Controls, n (%)**	**Cases, n (%)**	**Model A**	***P*** **value**	**Model B**	***P*** **value**	**Model C**	***P*** **value**
**Rs17817449**									
Co-dominant	TT	114 (77.0)	114 (77.0)	1.00		1.00		1.00	
	GT	33 (22.3)	30 (20.3)	0.91 (0.52–1.58)	0.74	0.001(0.00–0.85)	0.04^*^	0.03 (0.00–1.65)	0.08
	GG	1 (0.7)	4 (2.7)	3.93 (0.44–35.23)	0.22	/	/	/	/
G dominant	TT	114 (77.0)	114 (77.0)	1.00		1.00		1.00	
	GG+GT	34 (23.0)	34 (23.0)	1.00 (0.59–1.70)	1.00	0.001 (0.00–1.29)	0.06	0.03 (0.001–1.63)	0.08
G recession	TT+GT	147 (99.3)	144 (97.3)	1.00		1.00		1.00	
	GG	1 (0.7)	4 (2.7)	4.00 (0.45–35.79)	0.21	/	/	/	/
**Rs6567160**									
Co-dominant	TT	103 (69.6)	107 (72.3)	1.00		1.00		1.00	
	CT	42 (28.4)	38 (25.7)	0.88 (0.55–1.44)	0.62	0.81 (0.06–11.61)	0.88	0.52 (0.04–7.47)	0.63
	CC	3 (2.0)	3 (2.0)	0.98 (0.20–4.87)	0.98	/	/	/	/
C dominant	TT	103 (69.6)	107 (72.3)	1.00		1.00		1.00	
	CC+CT	45 (30.4)	41 (27.7)	0.89 (0.56–1.43)	0.63	0.89 (0.07–11.96)	0.93	0.56 (0.40–7.69)	0.66
C recession	TT+CT	145 (98.0)	145 (98.0)	1.00		1.00		1.00	
	CC	3 (2.0)	3 (2.0)	1.00 (0.20–4.96)	1.00	/	/	/	/

Model A: crude HR; Model B: Adjusted for age, sex, education, job, smoking, alcohol consumption, BMI, physical activity; Model C: Adjusted for age, sex, education, job, smoking, alcohol consumption, BMI, physical activity, hypertension, diabetes, and dyslipidemia. Statistical significance was defined as *P* < 0.025.

### Retrospective cohort study

A total of 13,967 participants without stroke were involved in this retrospective cohort study. Rs17817449 showed higher frequencies for BMI (*P* = 0.001) and diabetes (*P* = 0.04), but rs6567160 had higher frequencies for BMI (*P*<0.001) only in the genotype GG than in the genotype TT (Table 5).

**Table 5:** Baseline characteristic of rs17817449 and rs6567160 (*n* =13,967).

**Variable**	**rs17817449**				**rs6567160**			
	**GG**	**GT**	**TT**	***P*** **value**	**CC**	**CT**	**TT**	***P*** **value**
Number	*n* = 220	*n* = 3,253	*n* = 10,494		*n* = 325	*n =* 3,597	*n =* 10,045	
Age (years)	63.26 ± 6.97	64.24 ± 6.99	64.35 ± 7.12	0.06	64.88 ± 6.96	64.19 ± 7.07	64.33 ± 7.09	0.20
Sex, men, n (%)	47 (21.4)	773(23.8)	2,367(22.6)	0.31	77 (23.7)	793 (22.0)	2,317 (23.1)	0.42
**Education, n (%)**				0.64				0.69
Primary or below	81 (36.8)	1,229 (37.8)	4,069 (38.8)		120 (36.9)	1,365 (37.9)	3,894 (38.8)	
Middle school	123(55.9)	1,727 (53.1)	5,517 (52.6)		173 (53.2)	1,903 (52.9)	5,291 (52.7)	
College or above	16 (7.3)	297 (9.1)	908 (8.7)		32 (9.8)	329 (9.1)	860 (8.6)	
**Occupation, n (%)**				0.15				0.61
Manual	88(40.0)	1,480 (45.5)	4,879 (46.4)		158(48.6)	1,655 (46.0)	4,625 (46.0)	
Non-manual	76 (34.5)	1,136 (34.9)	3,594 (34.2)		107 (32.9)	1,215 (33.8)	3,484 (34.7)	
Other	56 (25.5)	637 (19.6)	2030 (19.3)		60 (18.5)	727 (20.2)	1,936 (19.3)	
**Smoking, n (%)**				0.14				0.56
Never	189 (85.9)	2,815 (86.5)	8,956 (85.3)		281 (86.5)	3,103 (86.3)	8,576 (85.4)	
Ever	12 (5.5)	227 (7.0)	724 (6.9)		18 (5.5)	233 (6.5)	712 (7.1)	
Current	19 (8.6)	211 (6.5)	814 (7.8)		26 (8.0)	261 (7.3)	757 (7.5)	
**Alcohol consumption, n (%)**				0.79				0.96
Never	88 (40.0)	1,260 (38.7)	4,166 (39.7)		127 (39.1)	1,423 (39.6)	3,964 (39.5)	
Ever	9 (4.1)	119 (3.7)	355 (3.4)		12 (3.7)	117 (3.3)	354 (3.5)	
Current	123 (55.9)	1,874 (57.6)	5,973 (56.9)		186 (57.2)	2,057 (57.2)	5,727 (57.0)	
**Physical activity**, ***IPAQ*****, n (%)**				0.19				0.90
Inactive	1 (0.5)	37 (1.1)	125 (1.2)		5 (1.5)	40 (1.1)	118 (1.2)	
Moderate active	29 (13.2)	604 (18.6)	1,845 (17.6)		58 (17.8)	653 (18.2)	1,767 (17.6)	
Active	190 (86.4)	2,612 (80.3)	8,524 (81.2)		262 (80.6)	2,904 (80.7)	8,160 (81.2)	
**BMI (Body mass index, kg/m** ^**2**^ **), n (%)**				0.001^*^				<0.001^*^
< 18.5	7 (3.2)	140 (4.3)	540 (5.1)		13 (4.0)	148 (4.1)	526(5.2)	
18.5–23.9	98 (44.5)	1,480 (45.5)	5,054 (48.2)		155 (47.7)	1,659 (46.1)	4,818 (48.0)	
24–27.9	78 (35.5)	1,233 (37.9)	3,759 (35.8)		103 (31.7)	1,351 (37.6)	3,616 (36.0)	
≥28	37(16.8)	400 (12.3)	1,141 (10.9)		54 (16.6)	439 (12.2)	1,085 (10.8)	
Hypertension, n (%)	111 (50.5)	1,550 (47.6)	4,962 (47.3)	0.62	154 (47.4)	1,729 (48.1)	4,740 (47.2)	0.66
Diabetes, n (%)	34 (15.5)	483 (14.8)	1,388 (13.2)	0.04^*^	53 (16.3)	519 (14.4)	1,333 (13.3)	0.08
Dyslipidaemia, n (%)	134 (60.9)	1,848 (56.8)	5,994 (57.1)	0.49	190 (58.5)	2,084 (57.9)	5,702 (56.8)	0.42
All stroke, n (%)	2 (0.8)	47 (1.4)	210 (2.0)	0.07	6 (1.8)	72 (2.0)	181 (1.8)	0.75
Ischaemic stroke, n (%)	1 (0.5)	22 (0.7)	108 (1.0)	0.14	3 (0.9)	33 (0.9)	95 (1.0)	0.99
Haemorrhagic stroke, n (%)	0 (0)	19 (0.6)	58 (0.6)	0.53	2 (0.6)	23 (0.6)	52 (0.5)	0.69
Unclassified stroke, n (%)	1 (0.5)	6 (0.2)	44 (0.4)	0.15	1 (0.3)	16 (0.5)	34 (0.3)	0.65

* *P* < 0.05.

**Figure 2:**
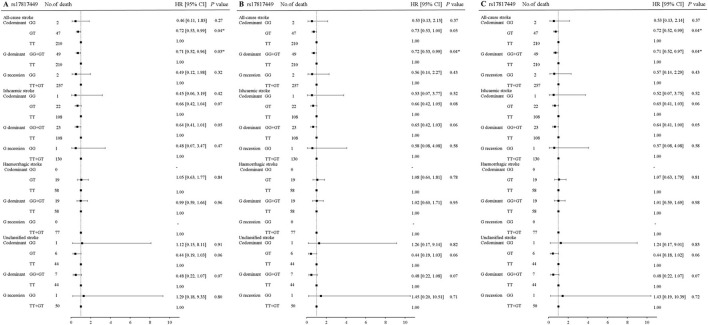
Association of rs17817449 at the FTO with the risk of fatal stroke in the participants of the GBCS (*n* = 13,967). **(A–C)** plots the hazard ratios and 95% confidence intervals for the three genotype models alongside the *P*-values. Model A: crude HR; Model B: adjusted for age, sex, education, job, smoking, alcohol consumption, BMI, physical activity; Model C: adjusted for age, sex, education, job, smoking, alcohol consumption, BMI, physical activity, hypertension, diabetes, and dyslipidemia. ^*^P < 0.05.

**Figure 3:**
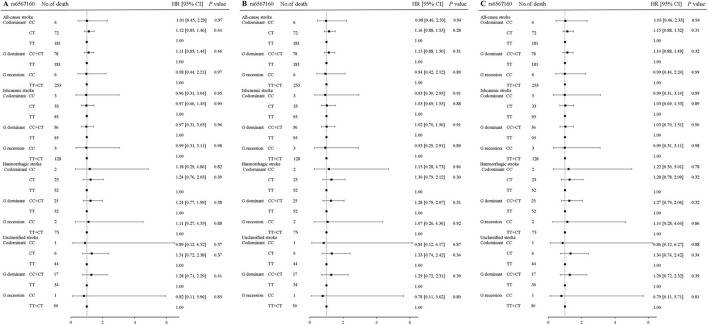
Association of rs6567160 at the MC4R with the risk of fatal stroke in the participants of the GBCS (*n* = 13,967). **(A–C)** plots the hazard ratios and 95% confidence intervals for the three genotype models alongside the *P*-values. Model A: crude HR; Model B: adjusted for age, sex, education, job, smoking, alcohol consumption, BMI, physical activity; Model C: adjusted for age, sex, education, job, smoking, alcohol consumption, BMI, physical activity, hypertension, diabetes, and dyslipidemia. ^*^P < 0.05.

In rs17817449 at the *FTO*, the genotypes GG+GT had a significant association with a decreased risk for fatal all strokes (aHR = 0.71, 95% CI 0.52–0.97, *P* = 0.04) and fatal ischemic stroke (aHR = 0.64, 95% CI 0.41–1.00, *P* = 0.05); the genotypes GT shared similar associations for fatal all strokes (aHR = 0.72, 95% CI 0.52–0.99, *P* = 0.04), fatal ischemic stroke (aHR = 0.65, 95% CI 0.41–1.03, *P* = 0.06); the genotypes GG shared insignificant associations for fatal all strokes (aHR = 0.53, 95% CI 0.13–02.14, *P* = 0.37) and fatal ischemic stroke (aHR = 0.52, 95% CI 0.071–3.75, *P* = 0.52), when the genotype TT was taken as a reference and a series of multiplicities were adjusted for age, sex, education, job, smoke, alcohol drinking, BMI, physical activity, hypertension, diabetes, and dyslipidemia (Figure 2). Moreover, significances were not observed in the associations of genotypes in rs6567160 with the risk of fatal stroke (Figure 3).

Table 6 shows the analyses stratified by hypertension, dyslipidemia, and physical activity. The genotypes GG+GT of rs17817449 decreased the risk of all fatal strokes in the participants with dyslipidemia (aHR = 0.63, 95% CI 0.39–1.00, *P* = 0.05) and without diabetes (aHR = 0.68, 95% CI 0.46–0.99, *P* = 0.049), although insignificances were observed for other subtypes of stroke. However, the genotypes CC+CT of rs6567160 were not associated with the risk of fatal stroke occurrence.

**Table 6:** Associations between rs17817449 and rs6567160 with the risk of stroke in stratified analyses on the G dominant model (*n* = 13,967).

	**rs17817449**						**rs6567160**				
**Stroke and variable**	**Genotype**		***P*** **value**	**Genotype**	***P*** **value**		**Genotype**		***P*** **value**	**Genotype**	***P*** **value**
	**TT**	**GG+GT**		**GG+GT**			**TT**	**CC+CT**		**CC+CT**	
**All strokes**										
Hypertension		No		Yes				No		Yes	
	1.00	0.63 (0.34–1.17)	0.14	0.75(0.52–1.07)	0.12		1.00	1.53 (0.94–2.49)	0.08	1.03(0.75–1.42)	0.85
Dyslipidemia		No		Yes				No		Yes	
	1.00	0.80 (0.52–1.22)	0.31	0.62 (0.39–0.99)	0.04^*^		1.00	1.18 (0.81–1.72)	0.38	1.11 (0.76–1.62)	0.59
Physical activity		Inactive		Moderate active				Inactive		Moderate active	
	1.00	0.58 (0.06–5.89)	0.65	0.70(0.39–1.26)	0.24		1.00	3.07 (0.60–15.60)	0.18	1.33 (0.79–2.24)	0.38
		Active						Active			
	1.00	0.72 (0.50–1.05)	0.09				1.00	1.06 (0.77–1.46)	0.71		
Diabetes		No		Yes–				No		Yes	
	1.00	0.68 (0.47–0.99)	0.045^*^	0.82(0.46–1.46)	0.50		1.00	1.13 (0.83–1.54)	0.45	1.18(0.69–2.00)	0.55
**Ischaemic stroke**										
Hypertension		No		Yes				No		Yes	
	1.00	0.42 (0.15–1.19)	0.10	0.74 (0.45–1.23)	0.25		1.00	1.27 (0.61–2.66)	0.52	0.74 (0.45–1.23)	0.25
Dyslipidemia		No		Yes				No		Yes	
	1.00	0.69 (0.36–1.29)	0.24	0.61 (0.32–1.17)	0.13		1.00	0.76 (0.42–1.38)	0.37	1.30 (0.78–2.18)	0.31
Physical activity		Inactive		Moderate active				Inactive		Moderate active	
	1.00	1.01 (0.07–14.64)	0.99	0.51 (0.21–1.22)	0.13		1.00	5.32 (0.35–80.14)	0.23	1.00 (0.47–2.14)	0.99
		Active						Active			
	1.00	0.71 (0.41–1.22)	0.21				1.00	0.94 (0.59–1.51)	0.81		
Diabetes		No		Yes				No		Yes	
	1.00	0.63 (0.36–1.09)	0.10	0.72 (0.33–1.58)	0.41		1.00	0.93(0.58–1.48)	0.76	1.28 (0.64–2.55)	0.48
**Haemorrhagic stroke**										
Hypertension		No		Yes				No		Yes	
	1.00	1.90 (0.69–5.26)	0.21	0.82 (0.44–1.52)	0.53		1.00	1.69 (0.61–4.67)	0.31	1.17 (0.68–2.01)	0.57
Dyslipidemia		No		Yes				No		Yes	
	1.00	1.36 (0.67–2.76)	0.40	0.72 (0.33–1.58)	0.42		1.00	1.79 (0.91–3.49)	0.09	0.72 (0.33–1.58)	0.42
Physical activity		Inactive		Moderate active				Inactive		Moderate active	
	1.00	–	–	1.33 (0.50–3.54)	0.56		1.00	/	/	1.64 (0.64–4.18)	0.30
		Active						Active			
	1.00	0.97 (0.52–1.81)	0.92				1.00	1.23 (0.70–2.16)	0.47		
Diabetes		No		Yes				No		Yes	
	1.00	0.97 (0.53–1.77)	0.92	1.05 (0.36–3.02)	0.93		1.00	1.30 (0.76–2.23)	0.34	1.12 (0.39–3.23)	0.83
**Unclassified stroke**										
Hypertension		No		Yes				No		Yes	
	1.00	0.31 (0.07–1.33)	0.11	0.61 (0.23–1.61)	0.32		1.00	2.02 (0.86–4.74)	0.11	0.92 (0.41–2.08)	0.84
Dyslipidemia		No		Yes				No		Yes	
	1.00	0.49 (0.17–1.43)	0.19	0.47 (0.14–1.58)	0.22		1.00	1.74 (0.81–3.72)	0.16	0.89 (0.35–2.27)	0.81
Physical activity		Inactive		Moderate active				Inactive		Moderate active	
	1.00	–	–	0.54 (0.11–2.53)	0.43		1.00	/	/	2.52 (0.75–8.49)	0.13
		Active						Active			
		0.45 (0.18–1.15)	0.09					1.10 (0.56–2.16)	0.79		
Diabetes		No		Yes				No		Yes	
	1.00	0.43 (0.17–1.11)	0.08	0.69 (0.14–3.35)	0.65		1.00	1.34 (0.70–2.55)	0.38	1.23 (0.31–4.86)	0.77

* *P* < 0.05.

*P* value was adjusted by age, sex, education, job, smoking, alcohol consumption, BMI, physical activity, hypertension, diabetes, and dyslipidemia.

## Discussion

We conducted a two-stage study to assess a series of associations between rs17817449 at the *FTO* and rs6567160 at the *MC4R* and the risk of stroke events. The results showed neither a significant association of the two loci with the risk of stroke occurrence nor an interaction between the two genetic loci known for obesity in a case-control study; the genotypes GG+GT of rs17817449 at the *FTO* showed a protective role in the risks of fatal all strokes and fatal ischaemic stroke, while no associations were observed for rs6567160 at the *MC4R* in a cohort study. To the best of our knowledge, this is the first study to address the relationships between the variants at the *FTO* and the *MC4R* and the risk of stroke events in relatively healthy middle-aged to older Chinese people.

The *FTO* and the *MC4R* are typical representatives of obesity, and more evidence supports the relationships between the two loci and stroke events. Our findings in a case-control study differ from those in a retrospective cohort study, wherein the former shows insignificant results. Like the Mannheim-Heidelberg stroke study in Germany (32), neither the *FTO* nor the *MC4R* was associated with LAA stroke in Han Chinese people (13). Taking into account the differences described above, we must elucidate the relationship between the genetic polymorphisms in obesity and the risk of stroke events in the following studies.

The genotypes GG+GT of rs17817449 at the *FTO* played a protective role in the risk of fatal stroke when the genotype TT was taken as a reference; in contrast, the genotype TT (major alleles) is a risk genotype in our study. However, the findings are contrary to previous studies in which the GG homozygote (minor alleles) of rs17817449 had a significantly increased risk of the acute coronary syndrome (ACS) in male Caucasians (33), which might link to regional and ethnic factors. For other genetic loci at the *FTO*, the genotype AA (minor alleles) of rs9939609 showed higher mortality rates of CHDs and cardiovascular diseases (CVDs) in Finnish (34), while rs9930609 was not significantly associated with a mortality risk of circulatory diseases in Danish males (35). A reasonable explanation for the different relationships between the *FTO* and stroke events should be a higher *fto* level in the brain, wherein the hypothalamus is located. A loss-of-function mutation in the *FTO* could impair hypothalamic development (3).

Upon further stratified analyses, the genotypes GG+GT of rs17817449 decreased the risk of all fatal strokes in populations with non-diabetes and dyslipidemia. Such correlations are reported first to date, although a series of studies showed a contradiction in rs17817449 on diabetes (36–39). Our results may be attributed to insulin resistance, the etiology of obesity, and an increased plasma leptin level (3, 38, 40). However, the rs17817449 was interestingly associated with a lipid profile but without obesity (36).

There are several limitations to this study. First, only two genetic loci were assessed; however, more polymorphisms at genetic loci known for obesity should be explored in stroke events. Second, the present study had a small sample size of cases, which limited an exact reflection on the underlying mechanism in genetic loci on stroke events. Third, the population density was too low in South China for the subjects to accurately represent all Chinese. Other cohorts and different ethnic populations should be further verified in the future. Nevertheless, we conducted a case-control study and a follow-up cohort study to provide evidence for the relationships between the *FTO* and stroke events, which is conducive to intervention in stroke occurrence earlier.

## Conclusion

Rs17817449 at *FTO*, but not rs6567160 at the *MC4R*, was associated with the risk of fatal all strokes; the genetic loci should be a potential biomarker for stroke events, and its functional role in stroke needs to be explored in relatively healthy middle-aged to older Chinese.

## Data availability statement

The original contributions presented in the study are included in the article/supplementary material, further inquiries can be directed to the corresponding author.

## Ethics statement

The study was approved by the Guangzhou Medical Ethics Committee of the Chinese Medical Association. All participants signed informed consent forms before participation. All methods in this study were performed in accordance with the Declaration of Helsinki. The patients/participants provided their written informed consent to participate in this study.

## Author contributions

Q-QZ contributed to this study for data collection and analysis. FZ contributed to the study design and wrote the manuscript. All authors reviewed the manuscript. All authors contributed to the article and approved the submitted version.

## Funding

This work was supported by the Guangzhou Municipal Science and Technology Project (201704030132, 202102080467) and the Guangdong Medical Research Foundation (A2022209). The funders had no role in the study design, data collection or analysis, or manuscript preparation.

## Conflict of interest

The authors declare that the research was conducted in the absence of any commercial or financial relationships that could be construed as a potential conflict of interest.

## Publisher's note

All claims expressed in this article are solely those of the authors and do not necessarily represent those of their affiliated organizations, or those of the publisher, the editors and the reviewers. Any product that may be evaluated in this article, or claim that may be made by its manufacturer, is not guaranteed or endorsed by the publisher.
